# Asthmatics Exhibit Altered Oxylipin Profiles Compared to Healthy Individuals after Subway Air Exposure

**DOI:** 10.1371/journal.pone.0023864

**Published:** 2011-08-29

**Authors:** Susanna L. Lundström, Bettina Levänen, Malin Nording, Anna Klepczynska-Nyström, Magnus Sköld, Jesper Z. Haeggström, Johan Grunewald, Magnus Svartengren, Bruce D. Hammock, Britt-Marie Larsson, Anders Eklund, Åsa M. Wheelock, Craig E. Wheelock

**Affiliations:** 1 Department of Medical Biochemistry and Biophysics, Division of Physiological Chemistry II, Karolinska Institutet, Stockholm, Sweden; 2 Division of Respiratory Medicine, Department of Medicine, Karolinska Institutet, Stockholm, Sweden; 3 Department of Entomology and Cancer Research Center, University of California Davis, Davis, California, United States of America; 4 Department of Public Health and Clinical Medicine, Respiratory Medicine and Allergy, Umeå University, Umeå, Sweden; 5 Division of Occupational and Environmental Medicine, Department of Public Health Sciences, Karolinska Institutet, Stockholm, Sweden; National Jewish Health, United States of America

## Abstract

**Background:**

Asthma is a chronic inflammatory lung disease that causes significant morbidity and mortality worldwide. Air pollutants such as particulate matter (PM) and oxidants are important factors in causing exacerbations in asthmatics, and the source and composition of pollutants greatly affects pathological implications.

**Objectives:**

This randomized crossover study investigated responses of the respiratory system to Stockholm subway air in asthmatics and healthy individuals. Eicosanoids and other oxylipins were quantified in the distal lung to provide a measure of shifts in lipid mediators in association with exposure to subway air relative to ambient air.

**Methods:**

Sixty-four oxylipins representing the cyclooxygenase (COX), lipoxygenase (LOX) and cytochrome P450 (CYP) metabolic pathways were screened using liquid chromatography-tandem mass spectrometry (LC-MS/MS) of bronchoalveolar lavage (BAL)-fluid. Validations through immunocytochemistry staining of BAL-cells were performed for 15-LOX-1, COX-1, COX-2 and peroxisome proliferator-activated receptor gamma (PPARγ). Multivariate statistics were employed to interrogate acquired oxylipin and immunocytochemistry data in combination with patient clinical information.

**Results:**

Asthmatics and healthy individuals exhibited divergent oxylipin profiles following exposure to ambient and subway air. Significant changes were observed in 8 metabolites of linoleic- and α-linolenic acid synthesized via the 15-LOX pathway, and of the COX product prostaglandin E_2_ (PGE_2_). Oxylipin levels were increased in healthy individuals following exposure to subway air, whereas asthmatics evidenced decreases or no change.

**Conclusions:**

Several of the altered oxylipins have known or suspected bronchoprotective or anti-inflammatory effects, suggesting a possible reduced anti-inflammatory response in asthmatics following exposure to subway air. These observations may have ramifications for sensitive subpopulations in urban areas.

## Introduction

Asthma is a common respiratory disorder associated with substantial morbidity, mortality and economic cost [1]. The prevalence of asthma is growing globally, particularly in urban areas where polluted air is a main trigger of asthmatic exacerbations. The pathogenesis of asthma in the urban environment can be affected by multiple air pollutants, including particulate matter (PM), ozone, and nitric oxides (NO_x_) [2], [3], [4]. In terms of PM, the most studied health effects to date are related to road emissions, which have been demonstrated to play an important role in asthma morbidity [5]. However, there are few studies focused on the health effects of other urban environments such as the subway system, which represents a major form of transportation in many cities. Even though the subway environment is a significant source of PM emission [6], [7], [8], [9], little is known about the biological effect(s) on the respiratory system. Given the potential for large-scale chronic exposure to subway air, and the cumulative risk for sensitive populations with compromised respiratory systems such as asthmatics, it is appropriate to examine the effects of this system in greater detail. Accordingly, the aim of this study was to investigate whether subway air can trigger acute inflammatory responses in asthmatics and healthy individuals.

Oxylipins are lipid mediators formed from unsaturated fatty acids via pathways involving dioxygen-dependent oxidation [10]. These compounds exert potent and often opposing effects upon multiple physiological processes, including inflammation and immunity [11], [12], [13], [14], [15], [16]. Three main enzymatic systems are active in oxylipin biosynthesis: cyclooxygenase (COX), lipoxygenase (LOX) and cytochrome P450 (CYP). Multiple oxylipins originating from arachidonic acid (also known as eicosanoids) are involved in asthma pathology (reviewed in [17]), including leukotrienes (LTs; *e.g.*, cysteinyl-LTs and LTB_4_), lipoxins (LXA_4_ and 15-epi-LXA_4_) and prostaglandins (PGE_2_ and PGD_2_) [11], [16]. The biological actions of oxylipins originating from the arachidonic acid (AA) cascade have been most extensively studied [11], [14], but the same enzymatic pathways can act on multiple unsaturated fatty acids (*e.g.*, linoleic- [LA] and docosahexaenoic acid [DHA]), which are potentially equally important for understanding disease pathology.

In this randomized crossover study, oxylipin levels were quantified in BAL-fluid from healthy individuals and mild asthmatics following exposure to Stockholm subway air and ambient air. Oxylipin profiles originating from multiple unsaturated fatty acid substrates from the COX, LOX and CYP biosynthetic pathways were measured in the lower respiratory tract, thus representing the first investigation of a number of pharmacologically interesting non-eicosanoid oxylipin mediators in human BAL-fluid. These data will be useful for: 1) improving our knowledge of the role of oxylipins in pro- and anti-inflammatory etiologies of asthma 2) understanding the mechanism of toxicity triggered by subway air and associated effects upon sensitive populations and 3) determining the basal levels of these important lipid mediators in healthy and asthmatic populations.

## Methods

### Subjects, Study Design and Sampling

Detailed descriptions of study design, exposure regimen, PM monitoring and the subjects are given elsewhere [18], [19]. In addition to the 18 healthy subjects [18], 15 mild intermittent asthmatics were included in the study [19] and the samples from both populations were analyzed simultaneously under the same conditions in randomized order. Hyperresponsiveness was tested in asthmatics and defined as ≥20% fall in forced expiratory volume in one second (FEV_1_) at an accumulated methacholine inhalation dose of <894 µg. Without challenge, asthmatics had normal lung function with FEV_1_ values within the same range as healthy individuals. β2-agonist use on demand was allowed throughout the study, but other medications including inhaled corticosteroids were not permitted. Participants were non-smokers, not habitual subway commuters and refrained from subway use at least 2 months prior to study initiation. Allergy tests were conducted on all subjects; 13 of the asthmatics and 2 of the healthy individuals indicated the presence of specific IgE antibodies against common inhaled allergens (Phadiatop®, Pharmacia-Upjohn, Uppsala, Sweden). The study was performed out of pollen season and airway infections were not allowed within 6 weeks before exposures. Gender, age and allergy test results are given in Table 1. All patients indicated on the provided study forms that non-steroidal anti-inflammatory drugs (NSAIDs) had not been taken prior to study initiation (except for patient 27, who indicated intake of an NSAID prior to one study event. No significant differences were observed in this individual relative to the other study participants).

**Table 1:** Diagnosis and allergy response for study cohort.

Diagnosis	Subject	Gender	Age	Allergy response^a^
**Asthmatic**	1	F	52	0
	4	F	40	0
	7	F	31	4
	12	F	27	1
	20	M	25	4
	21	M	25	5
	23	M	23	4
	24	M	22	5
	25	F	21	5
	26	F	21	6
	27	F	20	5
	28	F	20	5
	32	F	19	5
	33	F	18	3
	34	M	18	2
**Healthy**	2	M	46	0
	3	M	42	0
	5	F	36	0
	6	F	31	0
	8	F	29	0
	9	F	28	0
	10	F	27	0
	11	M	27	0
	13	M	26	0
	14	M	26	2
	16	M	25	0
	17	F	24	0
	18	M	24	0
	19	M	24	0
	22	M	22	0
	29	M	18	0
	30	M	18	1
	31	M	18	0

a Allergy response test values are rated from 0–6 (negative; 0: <0.35 kU/L, positive; 1: 0.35–0.69, 2: 0.7–3.4, 3: 3.5–17.4, 4: 17.5–49, 5: 50–99, 6: >99 kU/L; Phadiatop®, Pharmacia-Upjohn, Uppsala, Sweden). Allergy was tested against Timothy, Acari, Horse, Cat, Mugwort, Birch, Dog, Olive, Lichwort, Fish, Cladosporium. Maximum response level for each individual is indicated in the table.

Subway exposures were conducted during rush hour (4–6pm) at a central subway station and PM levels were monitored as previously described [18]. The exposure site consisted of a small room located a few meters below the platform level equipped with large double doors at both ends of the space used for the exposure (approximately 4 m apart), creating a space fully vented with the subway platform air. Mouth breathing was induced using an exercise bicycle in 15 min intervals, and resistance was set to normalize breathing rates to 20 L/min/m^2^ body surface for both test groups at the subway station (“Odenplan”) and in the ambient air environment.

The sampling time point 14 h following exposure was selected to enable comparison with a previous exposure study performed in a Stockholm road tunnel [20], and signifies the repair phase where the initial inflammatory mobilization in response to acute exposure normally is attenuated by anti-inflammatory mediators to return the system to base-line levels. Lung function was determined through spirometry prior to bronchoscopies. Five 50 ml aliquots of phosphate buffered saline (PBS; 37°C) were instilled in the middle lobe, gently aspirated and collected in a siliconized plastic bottle kept on ice. Recovered BAL-fluids were centrifuged at 400× *g* for 10 min at 4°C, to separate BAL-cells from supernatant. The cell pellet was resuspended in RPMI-1640 medium (Sigma-Aldrich, Irvin, UK) and the viability was determined by trypan blue exclusion. The study was approved by the Regional Ethical Review Board Stockholm with the case numbers 2006/643-31/4 (healthy individuals) and 2007/748-31/3 (asthmatics). All patients provided written informed consent.

### Oxylipin profiling

The oxylipin analysis methods have previously been published [21], [22] and are only briefly described here. Analytical standards and deuterated surrogates (Table S1) were obtained from either Cayman Chemical (Ann Arbor, MI, USA), Larodan Fine Chemicals AB (Malmö, Sweden), Biomol International (Plymouth Meeting, PA, USA) or synthesized in-house [21]. Oxylipins were extracted from 4 ml of BAL-fluid using Waters Oasis-HBL cartridges (Milford, MA, USA) preconditioned with wash solution (H_2_O∶MeOH; 95∶5, in 0.1% acetic acid). BAL-fluid aliquots, 200 µl wash solution, 10 µl of surrogate standards (400 nM/standard), 10 µl anti-oxidant and enzyme inhibitor solution (0.2 mg/ml of butylhydroxytoluene, ethylenediaminetetraacetic acid, thiamine pyrophosphate and iodomethacine) were applied on cartridge, rinsed with wash solution, eluted with 0.5 ml methanol followed by 1.5 ml ethylacetate and collected into polypropylene tubes containing 6 µl 30% glycerol in methanol. Solvent was stripped and the sample was re-suspended in 50 µl methanol containing the technical standard 1-cyclohexyl-dodecanoic acid urea (CUDA; 800 nM). Samples were then centrifuged and the supernatants stored at −20°C until analysis.

Oxylipin profiling was performed using 10 µl sample injections on an Agilent 1200SL separation module (Palo Alto, CA, USA) via a 2.0×150 mm Pursuit C18 column kept at 40°C (5 µm particle size; Varian, Lake Forest, CA, USA) coupled to an ABI QTRAP®4000 hybrid triple quadrupole/linear ion trap mass spectrometer (Foster City, CA, USA). The samples were kept at 4°C prior to injection. Oxylipins detected above the limit of quantification (LOQ) were quantified, recalculated back to the original BAL-fluid concentrations and normalized to BAL-recovery (V_[Recovered volume]_/V_[Instilled volume]_). Normalization to BAL-recovery did not affect the overall trends in the material as shown in (Figure S1). BAL-recovery values and BAL-recovery Subway/Control (S/C) ratios were not significantly different between the groups (p = 0.2) and (p = 0.6), respectively. No correlation between recovery and lung function (FEV_1_) was found (p = 0.5). Concentrations adjusted for BAL-recovery (Table S2), absolute concentrations (Table S3), and BAL-recovery information (Table S4) are provided in the Supporting Information.

### Immunocytochemistry

Approximately 60,000 BAL-cells/slide were cytocentrifuged onto SuperFrost®Plus glass slides (Menzel-Gläser, Braunschweig, Germany) at 450 rpm for 3 min for cell differential counts and further analysis by immunocytochemistry. Fluorescent immunocytochemistry was performed on the prepared cytospin slides with monoclonal antibodies against 15-LOX-1 (dilution 1∶1000, [23]), peroxisome proliferator-activated receptor gamma (PPARγ; dilution 1∶100, Santa Cruz sc-7196) produced in rabbit, and COX-1 (dilution 1∶200, Invitrogen 35–8100) and COX-2 (dilution 1∶200, Invitrogen 35–8200) produced in mouse. Cy5 labeled anti-rabbit sera from donkey (dilution 1∶1000, Amersham 711-175-152) and Alexa488 labeled anti-mouse sera from goat (dilution 1∶500–1∶1000, Molecular Probes A11001) were used as secondary antibodies. Normal goat serum was purchased from Vector Laboratories (Burlingame, CA, USA; S-1000 and S-4000) and normal donkey serum from Sigma-Aldrich (D9663). Before immunostaining, slides were fixed in 10% formalin and washed in PBS before antigens were retrieved by boiling the slides in citrate buffer for 30 min. Cell membranes were permeabilized by methanol at −20°C for 10 min, washed in 0.4% PhotoFlo (Photax K5010640) and air dried prior to rehydrating in PBS and blocking in 3% bovine serum albumin (BSA) for 30 min. Unspecific binding was blocked using antibody dilution buffer (ADB; 10% serum, 3% BSA, 0.05% TritonX in PBS) for 30 min. Samples were incubated in a dilution buffer of the primary antibody in a humidified chamber at 4°C for 16 h. The slides were washed in 1) PBS containing 0.4% PhotoFlo and 2) 0.01% TritonX, before being incubated for 1 h with the secondary antibody. After repeating the washing steps 1) and 2), the slides were counter-stained with 4′,6-diamidino-2-phenylindole, dihydrochloride (DAPI, Invitrogen D1306) and mounted with ProLong® Gold antifade reagent (Invitrogen P36930).

Visualization of the antigen-antibody complexes was performed using fluorescent microscopy, and images were acquired using Openlab (PerkinElmer, Waltham, MA, USA) and analyzed using the ImageJ freeware [24]. Total fluorescence intensity was measured for ≥100 cells in each slide from the same individuals as the oxylipin measurements (asthmatics n = 15, healthy individuals n = 16; insufficient patient material was available from 2 healthy individuals), background was subtracted and values were normalized to the corresponding total fluorescent DAPI staining of the corresponding cells in each slide. The choice of ≥100 cells for sampling was based upon common protocols in cytology as recently utilized by Gardner *et al.* [25]. The ≥100 quantified cells were randomly selected from on average 3 representative micrographs. Results are not reported for 3 healthy individuals due to a failure to pass internal quality controls.

### Statistical methods

Normalization for inter-individual variation was performed (*i.e.*, Subway/Control [S/C]), improving study power from 0.60 to 0.73. No multiple hypothesis testing correction was performed given that only 28 compounds were present above the LOQ, which at an α = 0.05 gives ∼1.4 potential false positives. Relative percent-composition was analyzed by grouping lipid metabolites according to class. Distribution analysis was performed using Shapiro-Wilks test (Analyse-it Software Ltd., Leeds, UK), and univariate statistics were performed using either Mann-Whitney or Student's t-test depending on the normality and homoscedastic nature of each variable (Prism, GraphPad Software, La Jolla, USA). Multivariate analyses by orthogonal projections to latent structures (OPLS) [26] were performed on S/C normalized data using SIMCA-P+ 12 (Umetrics AB, Umeå, Sweden) following mean centering and pareto scaling. For oxylipin data, only compounds above the LOQ were included in the analysis. Cell differential data were calculated as percent composition as previously reported in [18], [19] and protein data were based upon the normalized intensity. Model performance was reported as correlation coefficients (R^2^) and predictive performance based on seven-fold cross-validation (Q^2^).

## Results

### Oxylipin profiling

Sixty-four oxylipins representing 3 metabolic pathways (COX, LOX and CYP) were screened using LC-MS/MS analysis. Twenty-eight oxylipins were present above the method LOQ and an additional 16 oxylipins present above the method limit of detection (LOD) (Table S1). Oxylipin levels ranged over 1 pM to 8 nM (>10^3^; Table S2), with intra-group coefficients of variation (CV) of 27–170% (Table 2).

**Table 2:** Oxylipin concentrations and immunostaining data from 1) Healthy controls 2) Healthy individuals following subway air exposure 3) Asthmatic controls and 4) Asthmatics following subway air exposure.

		Healthy (H)				Asthmatics (A)				p-values^b^			
		Control (C)		Subway (S)		Control (C)		Subway (S)		Control	Subway	Healthy	Asthmatics
PUFA	Oxylipin^a^ (pM)	Average	CV(%)^c^	Average	CV(%)	Average	CV(%)	Average	CV(%)	H vs A	H vs A	C vs S	C vs S
AA^d^	5-HETE	199	52	170	44	306	107	240	84	0.2	0.2	0.3	0.4
	12-HETE	64	95	78	109	128	57	129	153	*0.01*	0.4	0.6	1.0
	15-HETE	672	129	666	83	1363	77	1089	99	*0.04*	0.2	1.0	0.3
	15-KETE	142	108	126	57	227	67	185	69	0.1	0.1	0.6	0.1
	LTB_4_	8	49	7	47	12	101	11	80	0.2	0.1	0.6	0.7
	6-*trans*-LTB_4_	47	55	39	56	44	92	38	86	0.8	0.9	0.2	0.5
	TXB_2_	64	43	60	42	66	54	63	67	0.9	0.8	0.5	0.7
	PGD_2_	15	96	15	68	32	108	39	148	0.1	0.1	0.9	0.6
	PGE_2_	10	48	13	87	15	50	11	89	*0.04*	0.7	0.4	0.1
	5(6)-EET	8	70	7	48	8	71	5	34	0.8	0.1	0.5	0.2
	8(9)-EET	7	72	4	58	7	46	6	59	0.7	0.1	*0.04*	0.3
LA^e^	9-HODE	202	103	423	138	188	49	128	44	0.8	0.05	*0.04*	*0.02*
	9-KODE	182	100	309	143	66	89	48	50	*0.02*	*0.02*	0.1	0.3
	13-HODE	1016	97	1458	111	1646	62	1151	85	0.1	0.5	0.2	0.1
	13-KODE	361	82	580	107	234	55	179	54	0.1	*0.01*	0.1	0.1
	EKODE	495	89	705	113	189	50	155	45	*0.01*	*0.01*	0.1	0.3
	9,10,13-TriHOME	262	58	312	81	193	66	116	47	0.2	*0.005*	0.4	0.1
	9,12,13-TriHOME	446	61	543	93	446	59	267	55	1.0	*0.04*	0.4	*0.04*
	9,10-DiHOME	408	38	448	44	310	51	267	77	0.1	*0.01*	0.3	0.3
	9(10)-EpOME	1877	28	1829	31	2012	48	2083	56	0.6	0.4	0.6	0.9
	12,13-DiHOME	476	41	517	50	373	53	322	75	0.1	*0.03*	0.3	0.3
	12(13)-EpOME	1938	27	1876	33	1976	44	2058	53	0.9	0.6	0.6	0.8
α-LA^f^	9-HOTE	9	129	20	161	11	48	9	55	0.5	0.2	0.1	0.1
	13-HOTE	52	100	72	116	91	62	71	100	*0.04*	1.0	0.2	0.3
EPA^g^	12-HEPE	9	101	13	67	16	99	11	108	0.1	0.6	0.1	0.4
	15-HEPE	64	132	68	74	125	89	93	84	0.1	0.3	0.9	0.2
DGLA^h^	15-HETrE	63	142	81	174	149	92	118	113	*0.03*	0.4	0.7	0.3
DHA^i^	17-HDoHE	278	137	301	95	461	72	379	93	0.2	0.5	0.9	0.4
ICC^j^	15-LOX-1	0.79	97	0.69	126	0.80	103	0.67	80	0.78	0.41	0.54	0.71
	COX-1	1.64	47	1.38	59	1.05	69	1.19	37	*0.03*	0.82	0.31	0.32
	COX-2	1.92	40	1.96	37	1.79	47	1.70	51	0.65	0.23	0.96	0.46
	PPARγ	0.55	43	0.73	89	0.60	54	0.55	63	0.93	0.29	0.80	0.41

a Oxylipin levels were normalized to the BAL-volume recoveries as described in Table S2 and Table S4,

b p-values are based on unpaired for A vs H and paired Student's T-test for C vs S, respectively. Values of p<0.05 are italicized,

c Coefficients of variation (%),

d Arachidonic acid,

e Linoleic acid,

f α-Linolenic acid,

g Eicosapentaenoic acid,

h Dihomo-γ-linolenic acid,

i Docosahexaenoic acid,

j Immunocytochemistry, RFU values normalized to DAPI intensities.

**Figure 1:**
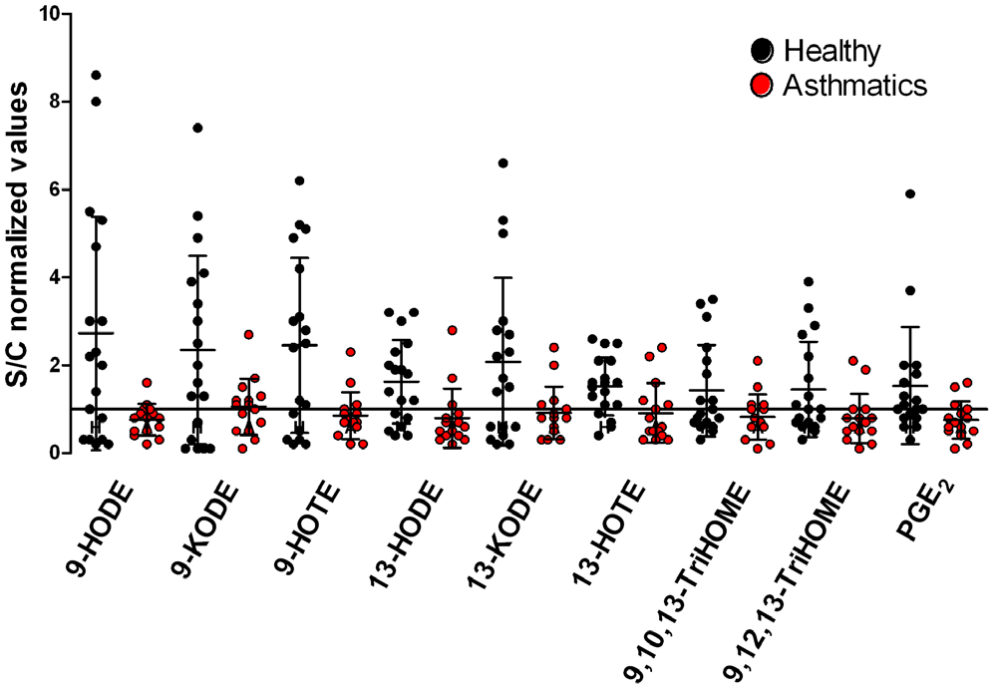
Alterations in oxylipin levels in response to subway air exposure. Significant differences (p<0.05) in baseline normalized oxylipin levels (Subway/Control, S/C) for healthy individuals (n = 18) and asthmatics (n = 15) following exposure to subway air. Values >1 indicate an increase in concentration following subway air exposure, and values <1 indicate a decrease.

**Figure 2:**
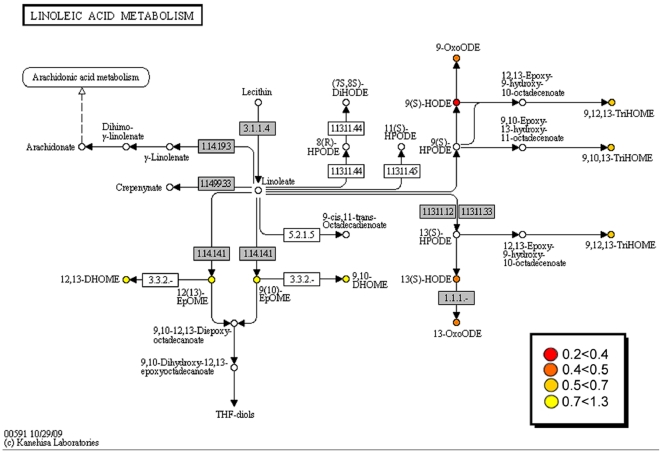
Alterations in oxylipin responses to subway air exposure mapped to the KEGG Linoleic Acid pathway (hsa00591). Differences between groups are shown as ratios (asthmatics/healthy individuals) between self-normalized oxylipin levels (Subway/Control, S/C; Table 3). Similar enzymatic conversions can occur via the α-linolenic acid pathway, but are not shown here (see KEGG pathway hsa00592). Data are color-coded as described in the figure to display the ratio and were mapped to KEGG using the KegArray software as previously described [58].

Nine oxylipins were significantly altered between asthmatics and healthy individuals in response to subway air exposure (Table 3, Table S5): 9- and 13-hydroxyoctadecadienoic acid (HODE), 9- and 13-oxooctadecadienoic acid (KODE), 9,10,13- and 9,12,13-trihydroxyoctadecenoic acid (TriHOME), 9- and 13-hydroxyoctadecatrienoic acid (HOTE), and PGE_2_ (p<0.05). The S/C ratio of all 9 compounds evidenced an increase in the healthy individuals following subway air exposure, whereas the asthmatics exhibited little change or a decrease (Figure 1). All 9 oxylipins originate from either the LA or α-linolenic acid (α-LA) pathways, with the exception of PGE_2_, which is a COX product of AA. In addition, the majority of these oxylipins are synthesized via the 15-LOX pathway as shown via their mapping to the KEGG pathway for LA metabolism (Figure 2).

**Table 3:** Response to subway air exposure normalized to individual baseline levels (Subway/Control) in oxylipins and immunostaining data^a^.

		Healthy		Asthmatics		
PUFA	Oxylipin	Average	CV(%)	Average	CV(%)^b^	p-values^c^
AA^d^	5-HETE	1.0	46	1.0	70	0.8
	12-HETE	1.6	96	1.1	165	0.4
	15-HETE	1.5	82	1.0	138	0.3
	15-KETE	1.2	62	1.0	115	0.7
	LTB_4_	1.1	50	1.2	75	0.7
	6-*trans*-LTB_4_	0.9	48	1.0	58	0.6
	TXB_2_	1.0	33	0.9	58	0.7
	PGD_2_	1.5	100	1.0	113	0.3
	PGE_2_	1.5	88	0.7	58	*0.03*
	5(6)-EET	1.1	55	0.9	65	0.3
	8(9)-EET	0.8	71	1.0	71	0.4
LA^e^	9-HODE	2.7	98	0.8	47	*0.01*
	9-KODE	2.3	91	1.0	60	*0.02*
	13-HODE	1.6	59	0.8	86	*0.01*
	13-KODE	2.1	92	0.9	63	*0.02*
	EKODE	2.1	88	1.1	84	0.1
	9,10,13-TriHOME	1.4	73	0.8	63	*0.04*
	9,12,13-TriHOME	1.5	74	0.8	71	*0.03*
	9,10-DiHOME	1.2	50	0.9	45	0.07
	9(10)-EpOME	1.1	55	1.3	68	0.4
	12,13-DiHOME	1.2	50	0.9	43	0.1
	12(13)-EpOME	1.1	53	1.3	65	0.4
α-LA^f^	9-HOTE	2.5	82	0.9	62	*0.004*
	13-HOTE	1.5	44	0.9	75	*0.01*
EPA^g^	12-HEPE	1.8	70	1.0	156	0.1
	15-HEPE	1.6	80	0.9	133	0.1
DGLA^h^	15-HETrE	1.8	125	1.0	151	0.2
DHA^i^	17-HDoHE	1.4	69	1.0	125	0.3
ICC^j^	15-LOX-1	1.1	77	1.6	89	0.4
	COX-1	1.1	109	1.9	93	0.05
	COX-2	1.1	31	1.2	79	0.4
	PPARγ	1.4	65	1.0	43	0.2

a Oxylipin levels were normalized to the BAL-volume recoveries as described in Table S2 and Table S4,

b Coefficients of variation (%),

c p-values are based on unpaired statistics with values of p<0.05 italicized,

d Arachidonic acid,

e Linoleic acid,

f α-Linolenic acid,

g Eicosapentaenoic acid,

h Dihomo-γ-linolenic acid,

i Docosahexaenoic acid,

j Immunocytoochemistry, RFU values normalized to DAPI intensities.

The absolute concentrations of several of these 9 oxylipins were also significantly different (p<0.05, Table 2). Control levels of PGE_2_ and 13-HOTE were significantly higher in asthmatics compared to healthy individuals, in contrast to 9-KODE, which was significantly lower (Figure S2). Following subway air exposure, 5 oxylipins from LA were significantly lower in asthmatics (9-HODE, 9- and 13-KODE, 9,10,13- and 9,12,13-TriHOME). For asthmatics, levels of 9-HODE and 9,12,13-TriHOME decreased following exposure to subway air. Interestingly, only 9-HODE evidenced significant shifts in both healthy individuals and asthmatics following exposure to subway air, but the vector of the change was opposite for the 2 groups, with an increase in healthy and a decrease in asthmatics.

At baseline levels, the 15-LOX-produced 12- and 15-hydroxyeicosatetraenoic acid (12- and 15-HETE) as well as 15-hydroxyeicosatrienoic acid (15-HETrE) were significantly higher and EKODE was significantly lower in asthmatics compared to healthy individuals (Figure S2). Following subway air exposure, EKODE as well as 9,10- and 12,13-dihydroxyoctadecenoic acid (DiHOME) were significantly lower in asthmatics compared to healthy individuals. In healthy individuals, 8(9)-epoxyeicosatrienoic acid (EET) was significantly decreased after subway air exposure.

**Figure 3:**
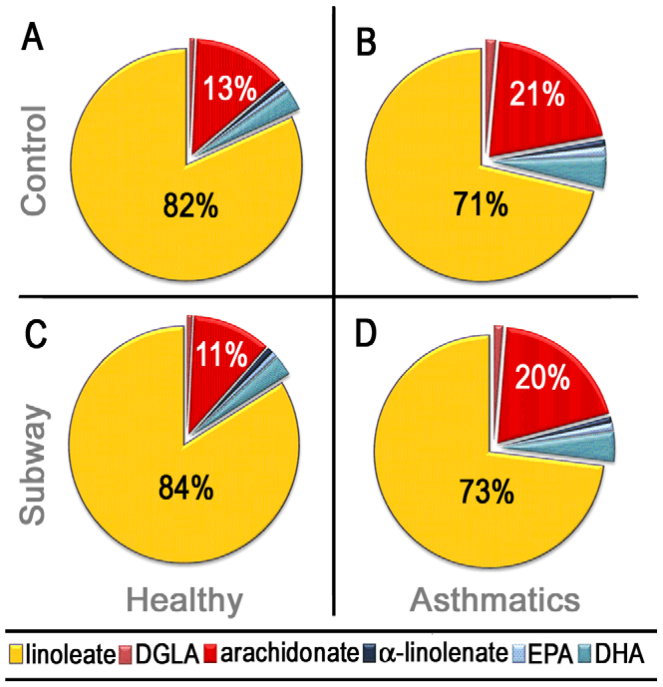
BAL-fluid oxylipin composition among patient groups. Following exposure to control air, healthy individuals (A) and asthmatics (B) evidenced significant differences in oxylipin levels on a fatty acid class-specific basis for linoleates (LA; p = 0.004), arachidonates (AA; p = 0.008), docosahexaenoates (DHA; p = 0.045), dihomo-γ-linolenates (DGLA; p = 0.007), α-linolenates (α-LA; p = 0.035) and eicosapentaenoates (EPA; p = 0.045). Following exposure to subway air, only LA (p = 0.022) and AA (p = 0.013) were significantly different between healthy individuals (C) and asthmatics (D). No significant changes were observed in healthy individuals or asthmatics following exposure to subway air, relative to exposure to control air; (A) vs (C) and (B) vs (D), respectively. The unlabeled fatty acids (DGLA, α-LA, EPA and DHA) are all present at <5% and are presumably more representative of a lack of coverage in the analytical method rather than potential biological differences in relative abundance.

To facilitate comparisons of global shifts in lipid metabolites, oxylipin levels were summed upon the basis of the unsaturated fatty acid substrate (*e.g.*, AA). On a lipid class percent composition basis, BAL-fluid was highly enriched in LA-derived metabolites, representing ∼70–80% of the overall oxylipin content as compared to ∼10–20% of AA metabolites. Other analyzed fatty acid classes, including DHA and eicosapentaenoic acid (EPA), were only represented by a few oxylipins in the analytical method. All fatty acid classes examined evidenced significant differences between healthy individuals and asthmatics at baseline levels (p<0.05; Figure 3A–3B). In contrast, significant differences were only observed in the linoleate and arachidonate oxylipin species following exposure to subway air (Figure 3C–3D). No differences were observed between the control and subway air exposure profiles for either asthmatics or healthy individuals.

Of the individual oxylipins, the proportion of the LA epoxide metabolites 12(13)-epoxyoctadecenoic acid (EpOME) and 9(10)-EpOME, and their corresponding diols 12(13)- and 9(10)-DiHOME, together represented ∼40–50% of total detected oxylipins in BAL-fluid (Figure S3). Other major BAL-fluid components were 13-HODE and 15-HETE, representing ∼10–15% each of the overall profiles. Thus, the majority of the oxylipin content in BAL-fluid is represented by a small number of high abundance metabolites.

### Immunocytochemistry

**Figure 4:**
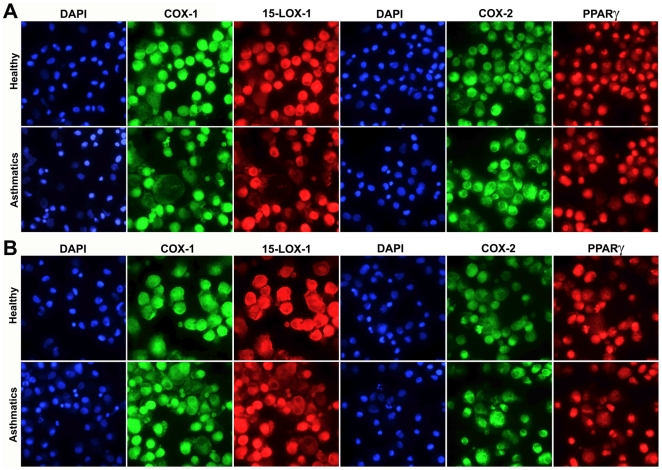
Fluorescent immunocytochemistry was performed on cytospun BAL-cells using monoclonal antibodies against COX-1, COX-2, 15-LOX-1 and PPARγ coupled with secondary antibodies labeled with fluorescent dyes Alexa488 (green) and Cy5 (red). Nuclear counter-staining was performed using DAPI (blue). Representative micrographs from a healthy and an asthmatic individual are shown for both the control air (A) and subway air (B) exposures. Semi-quantitative evaluation of staining intensities was performed using ImageJ software [24].

**Figure 5:**
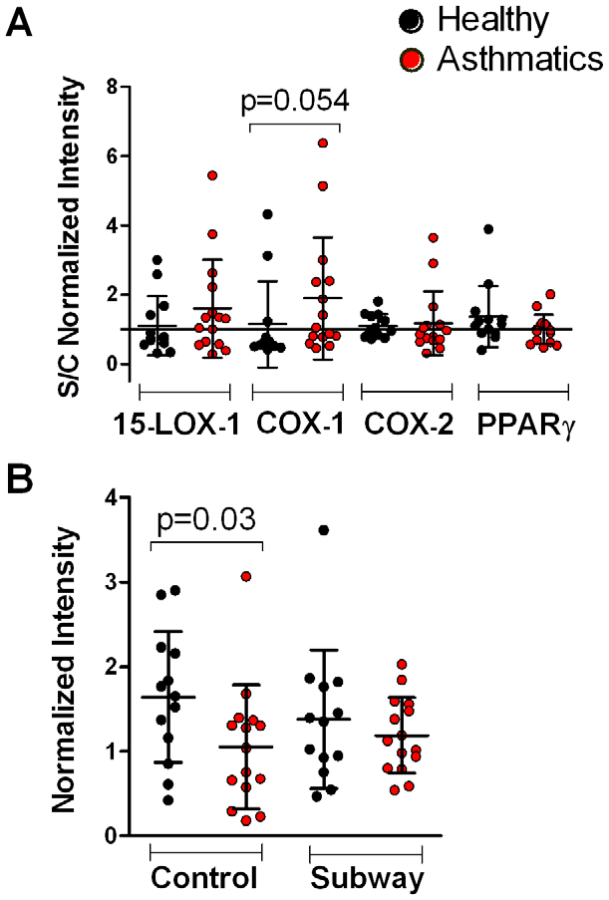
Semi-quantitative analysis of immunostained cytospun BAL-cells. (A) Baseline normalized expression levels (Subway/Control, S/C) for immunostaining of BAL-cells revealed an intensified response in asthmatics compared to healthy individuals for COX-1 (p = 0.054). Values >1 indicate an increase in concentration following subway air exposure and values <1 indicate a decrease. (B) Analysis of the subway air and control exposures as separate groups gave significantly lower COX-1 levels in asthmatics compared to healthy individuals at baseline levels (p = 0.03).

Representative micrographs of the immunostaining of BAL-cells against COX-1, COX-2, 15-LOX-1 and PPARγ are shown in Figure 4. Significant alterations were observed for COX-1 (Table 2), with levels following ambient air exposure higher in healthy individuals relative to asthmatics (p = 0.03, Figure 5B); however no differences were observed following exposure to subway air. COX-1 staining approached significance (p = 0.054) when evaluating the overall response to subway air exposure (*i.e.*, S/C-normalized values) for asthmatics versus healthy individuals (Figure 5A). No significant differences were observed for COX-2, 15-LOX-1 or PPARγ (Table 3).

### Multivariate modeling

**Figure 6:**
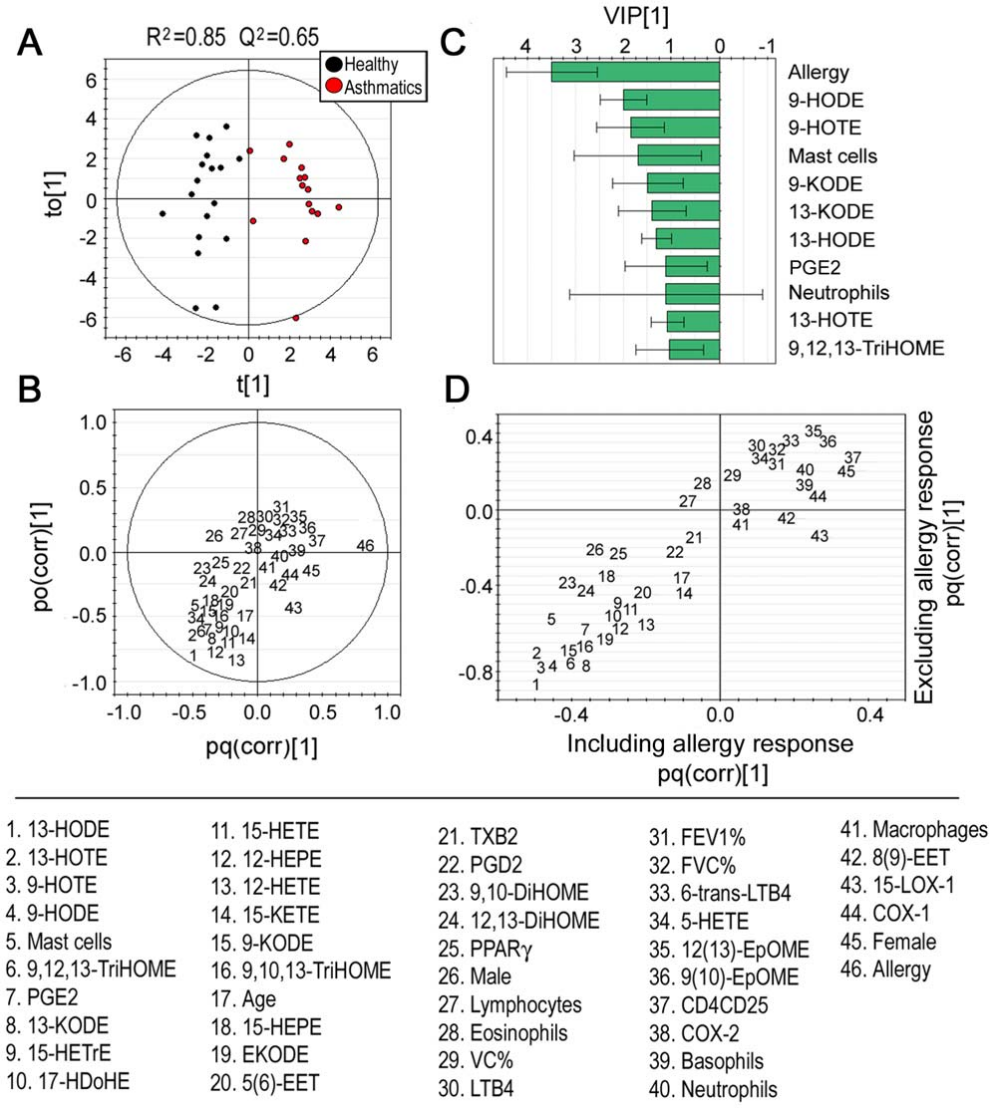
OPLS analysis integrating oxylipin, immunocytochemistry and clinical variables, with asthma diagnosis as the sole response variable. The scores plot (A) shows a robust separation of asthmatics (red dots) and healthy individuals (black dots) into 2 distinct groups. The loading (B) and VIP plots (C) indicate that in addition to the allergy response variable, the 12/15-LOX metabolites are driving the separation of the 2 groups. Error bars indicate 95% confidence interval. The SUS plot (D) correlates OPLS models that include (x-axis) and exclude (y-axis) the allergy response variable, revealing a high level of correlation between the models, suggesting that removal of the allergy response variable does not affect the model contribution of the remaining variables. Oxylipin abbreviations are as described in the text and Table S1, VC = vital capacity, FVC = forced vital capacity, and FEV_1_ = force expiratory volume in 1 sec.

Multivariate OPLS modeling investigating the overall trends in the integrated oxylipin, immunocytochemistry, and clinical data (including previously reported BAL-cell differential and T-cell activation marker data [18], [19]) resulted in a robust separation of asthmatics and healthy individuals (R^2^Y(cum) = 0.85, Q^2^(cum) = 0.65; Figure 6). As indicated in the loading- and variable importance in projection (VIP) plots (Figure 6B–6C), the single most important variable for driving the separation between groups was allergy response. This variable is constructed from the maximal Phadiatop® response observed in each individual, and thus reflects the *degree* of atopy for the individual rather than the *presence* of atopy. Removing this variable from the OPLS analysis weakened the model, particularly in terms of predictive power (R^2^Y(cum) = 0.30, Q^2^(cum) = 0.16). However, it also removed all significant orthogonal vectors, indicating that the intensity of the allergy response is the main contribution factor to the orthogonal variance in the data set. Removing the allergy variable did not change the overall trends in the OPLS as indicated by the Shared and Unique Structures (SUS) plot in Figure 6D. The 9 oxylipins identified as significantly altered via univariate testing were the main contributing variables to the separation between healthy individuals and asthmatics following allergy response (Figure 6B–6C), thus providing further evidence that they are not false positives resulting from multiple hypothesis testing. None of the variables derived from the immunostaining of COX-1, COX-2, 15-LOX-1 or PPARγ made major contributions. Furthermore, no correlations between specific cell types and oxylipins were observed. However, the CD4+ T-cells expressing the activation marker CD25 were associated with asthmatics, indicating an inflammatory response in these individuals in response to subway air exposure. The prominence of the mast cell variable should be interpreted with caution, as the majority of the measurements for this cell type were close to the LOD, and below the LOQ [18], [19].

## Discussion

The subway is a primary means of transportation in metropolitan areas worldwide. In spite of recent reports of significant toxicological effects of subway emission *in vitro* [27], [28], few studies have investigated the potential adverse effects of exposure to this environment *in vivo* [7], [18], [19], [29], [30], particularly in terms of sensitive subpopulations with respiratory disease. In this study, we investigated the acute effects of exposure in the Stockholm subway during rush hour traffic in mild intermittent asthmatics and healthy individuals. To provide a quantitative measure of subway air responses in the distal lung, the profiles of pro- and anti-inflammatory oxylipins were studied in BAL-fluid. In spite of being important in the complex interplay that regulates inflammatory response and subsequent resolution, few of these compounds have been investigated in the context of human lung disease.

The trend for the 9 oxylipins found to be significantly altered between asthmatics and healthy individuals was identical, with exposure to subway air eliciting increases in the relative oxylipin levels in healthy individuals, and decreases or no change in asthmatics (Figure 1). Of these mediators, the COX product of AA, PGE_2_ is the most well studied with both positive and negative effects in asthma pathology (reviewed in [15]). The mediator has a demonstrated bronchodilatory effect with inhibition of both early and late phases, and mouse models indicate that COX-1 activity has a predominately bronchoprotective effect. On the contrary, COX-2 is mainly pro-inflammatory by causing infiltration of inflammatory cells in the lung [31], [32]. Accordingly, PGE_2_ can exert diverse effects on the human lung that most likely are regulated by different pathways. In this study, immunocytostaining results for COX-1 showed significantly higher baseline expression levels in healthy individuals relative to asthmatics (Figure 5B), which lost significance following exposure to subway air. This trend in COX-1 abundance in BAL-cells did not, however, follow that of the observed PGE_2_ levels (Table 2). Furthermore, the multivariate modeling did not reveal any correlations between COX-1 and oxylipin levels (Figure 6), suggesting that the elevated COX-1 levels in BAL-cells may not be directly of relevance to the observed PGE_2_ alterations.

The other 8 oxylipins with significantly different responses following subway air exposure are metabolites originating from LA or α-LA. These mediators are all from the 15-LOX pathway (Figure 2) and clustered together in the multivariate modeling, further suggesting that they may be co-regulated (Figure 6). The observation that these oxylipins were the main driving variables for the OPLS model, regardless of whether the allergy response was included in the model (Figure 6D), further stresses their importance in differentiating the biological response between asthmatics and healthy subpopulations. The human variant of the 15-LOX enzyme in monocytes and alveolar macrophages can be induced by interleukins associated with asthma, including IL-4 and IL-13 (reviewed in [33]). Accordingly, the prevalence of 15-LOX-1 in BAL-cells was examined via immunocytochemistry. However, no alterations were observed in 15-LOX-1 levels in BAL-cells between healthy individuals and asthmatics following exposure to either ambient or subway air (Figure 4 and Figure 5). The lack of alterations in 15-LOX-1 expression is also in concordance with previous studies in asthmatics [34]. The observed increase of 15-LOX metabolites in healthy individuals may consequently be derived from other cell types in the lung. Several cell types in the respiratory tract possess the potential to express 15-LOX-1, including bronchial epithelial cells, alveolar macrophages, eosinophils, and mast cells, with epithelial cells evidencing the greatest levels of 15-LOX-1 [35], [36]. It is therefore likely that the observed shifts in oxylipin levels are due to 15-LOX activity in epithelial cells.

Among the significantly altered 15-LOX products, 13-HODE is the most well studied LA derivative and of demonstrated importance in airway disease [33], [37]. 13-HODE has been shown to alter the activity of the anti-inflammatory receptor PPARγ in a concentration dependent manner [33], [38], [39]. PPARγ has been proposed to be closely involved in regulating the inflammatory states underlying many airway diseases [40], [41] and PPARγ agonists have been suggested as alternative anti-inflammatory therapeutics in asthma [42]. Three other linoleate metabolites with demonstrated binding affinity to PPARγ, 9-HODE, 9-KODE and 13-KODE [38], [43], [44], were also significantly altered between asthmatic and healthy individuals, and main driving variables in the multivariate model (Figure 6). However, no alterations in PPARγ protein expression were found in BAL-cells (Figure 4 and Figure 5). The other LA-derived oxylipins, the TriHOMEs, have not been extensively studied in the literature and little is known about their biological function. Given the observed shifts in their levels following exposure to subway air, as well as in other studies in our group (data not shown), this group of compounds warrants further investigation.

Several studies have suggested that high 15-LOX activity and the levels of the AA 15-LOX product 15-HETE are mainly indicative of pro-inflammatory responses in asthma [34], [36], [45]. In this study, the concentrations of 15-HETE were significantly greater in asthmatic baseline levels (Table 2 and Figure S2), but not following subway air exposure. This difference could be suggestive of healthy individuals having lower lung inflammatory activity at baseline levels, which could also explain why subway air appears to elicit a more active response in the healthy individuals.

To date, little is known of the global oxylipin metabolic composition of BAL-fluid in human subjects. At baseline levels, profiles of all fatty acid classes in BAL-fluid were significantly different when analyzed on a percent composition basis (Figure 3). Particularly the ratios of LA∶AA metabolites between asthmatics and healthy individuals were different, with healthy individuals evidencing a LA∶AA ratio of 6.3 relative to 3.4 in asthmatics (Figure 3). The biological significance of these compositional differences is unclear, but exposure to subway air had little effect upon the overall metabolic LA∶AA ratios (7.6 and 3.7 for healthy individuals and asthmatics, respectively). The higher prevalence of linoleates in healthy individuals compared to asthmatics is contrary to the mounting hypothesis of high LA consumption as a risk factor for developing allergic diseases, including asthma [46]. This theory is however based on the abundance of the LA precursor, which can be readily converted to AA *in vivo* via the Δ6-desaturase prior to further metabolism to the AA-derived oxylipins. In addition, in a cystic fibrosis model, both *in vitro* and *in vivo* studies reported that LA supplementation increased the level of AA-derived pro-inflammatory oxylipins [47]. Accordingly, these observations suggest that any manifested biological effects are most likely due to small changes in specific compounds rather than overall lipid classes.

The results in Figure 1 and Figure 3 show a clear predominance of LA-derived relative to AA-derived oxylipins, both in terms of overall abundance as well as shifts following subway air exposure. These observations can be explained in terms of enzyme substrate specificity and relative abundance of the individual fatty acid precursors. In contrast to COX-1 and COX-2, which have preferred substrate specificity for AA [10], [48], [49], 15-LOX uses both LA and AA as substrate [50]. However, LA is the preferred substrate for 15-LOX *in vitro* [51] as well as in human tracheobronchial epithelial cells [52]. In lung tissue from healthy individuals, AA and LA amounts have been reported in 1∶1 ratios [53]; however in human bronchial epithelial cells (the likely source of the oxylipins quantified in this study) the levels of AA are ∼2/3 of LA [54]. Accordingly, for data interpretation it is important to consider both the relative fatty acid levels in different cellular environments as well as the enzyme substrate specificity.

Subway air has a unique composition relative to other sources of urban air pollutants both in terms of chemical composition and particulate size [55]. Several *in vitro* studies have indicated that PM from subway air is more genotoxic than PM from the street level [27], [28]. In contrast to PM from road emission consisting primarily of combustion-related PM, subway PM is formed from wear and tear of rails and train brakes. This results in a concomitant high iron content, which can act as catalyst and cause oxidative stress through redox cycling [27]. In comparison to a previous road tunnel air exposure study performed with the same study design and sampling equipment, the current study represented comparable levels of PM_10_ (242±40 µg/m^3^) and PM_2.5_ (77±10 µg/m^3^) [18], [20], which is in rough agreement with previously published work [6]. In spite of the similarities in PM exposure levels, these environments elicited distinctly different inflammatory responses in healthy individuals. In particular, the significant increases in the number of inflammatory cells and cytokines observed following road tunnel air exposure were not observed following subway air exposure [18], [19]. The oxylipin results do however tell a different story, indicative of a clear response following subway air exposure in spite of the absence of classical inflammatory indicators. Arguably, the observed differences in responses between asthmatics and healthy individuals may be the result of airway constriction in the asthmatics, which could alter dose or site of impact of the exposure. However, close monitoring of the lung function throughout the study, including peak expiratory flow (PEF), spirometry, and exhaled NO [18], [19], showed that subway air exposure did not elicit any airway constriction. As such, the lack of alterations in oxylipin levels in asthmatics is not likely to be an effect of altered airflow patterns. In contrast, the previously reported increase in CD4+CD25+ cells in asthmatics, but not in healthy subjects, indicates that the asthmatics have an ongoing inflammatory response that is absent in healthy individuals. Conversely, an increase in regulatory T-cells (Treg; FoxP3+CD4+CD25+) in healthy, but not in asthmatic subjects following subway air exposure is indicative of an ongoing anti-inflammatory response in healthy individuals that is lacking in asthmatics. As such, the biological activity of the observed oxylipins may have an attenuating effect on inflammation. It is noteworthy that a single exposure well below levels classified as harmless to workers in the New York [7], [56] and London [8] subway systems, elicits such a divergent response in healthy and asthmatic populations.

The data presented here are potentially suggestive of an overall defensive response in healthy individuals following exposure to subway air that is not observed in asthmatics. This observation could either be an indication of a decreased initial acute response following exposure to subway PM similar to that observed following exposure to diesel exhaust [57], or an indication of a hampered ability to attenuate the initial acute inflammation among asthmatic subjects. The relative importance of the previously reported increase of activated T-cells in the asthmatic group following subway exposure in the multivariate models (Figure 6D), in conjunction with previously reported increases in Treg cells in healthy individuals, but not asthmatics, following subway air exposure are indicative of the latter [18], [19]. Furthermore, the relative oxylipin profiles are suggestive of involvement of the 15-LOX pathway in these processes, which may be relevant to disease etiology and the overall pathology of asthma.

### Conclusion

In this study distinct fluctuations in oxylipin levels were observed, suggesting that subway air can trigger responses in the human lung in a pathology-dependent manner. In particular, these data show that asthmatics possess different oxylipin profiles compared to healthy individuals, both at baseline levels and in response to exposure to Stockholm subway air. This study is the first to report metabolic profiling of oxylipin levels in human BAL-fluid, with results suggesting that this matrix may provide a sensitive tool for studying subtle inflammatory responses in the airway that are not detectable with classical inflammatory markers. The distinct responses in oxylipin profiles between healthy individuals and asthmatics suggests that these components are important in asthma pathology, and their use for monitoring purposes as well as treatment should be further examined.

## Supporting Information

Figure S1 **Coefficients of variance (CVs) for all oxylipins before and after BAL-fluid recovery volume normalization (norm).** These data support the decision to normalize the oxylipin data with the BAL-fluid recovery volumes due to a decrease in the observed CVs. Abbreviations: AS: Asthmatics following subway air exposure; AC: Asthmatics following control air exposure; HS: Healthy individuals following subway air exposure; HC: Healthy individuals following control air exposure.
(TIF)

Figure S2 **Oxylipin baseline concentrations (pM) of those metabolites indicating significantly different levels between healthy individuals and asthmatics.**
(TIF)

Figure S3 **Oxylipin composition in BAL-fluid of the 4 different exposure groups.** Oxylipin naming is as provided in Table S1, with data taken from Table 2.
(TIF)

Table S1 **Detected and quantified oxylipins as well as oxylipin nomenclature.**
(XLS)

Table S2 **Individual oxylipin concentrations normalized for BAL-fluid recoveries (pM).**
(XLS)

Table S3 **Absolute oxylipin concentrations (pM).**
(XLS)

Table S4 **BAL-fluid recovery volumes for bronchoscopies.**
(XLS)

Table S5 **Individual values for Subway/Control (S/C) normalization of oxylipin levels.**
(XLS)
