# Supplementary figures and images for: Asthmatics Exhibit Altered Oxylipin Profiles Compared to Healthy Individuals after Subway Air Exposure
Source: PLoS One. 2011 Aug 29;6(8):e23864. doi: 10.1371/journal.pone.0023864 (PMC3163588; doi:10.1371/journal.pone.0023864)

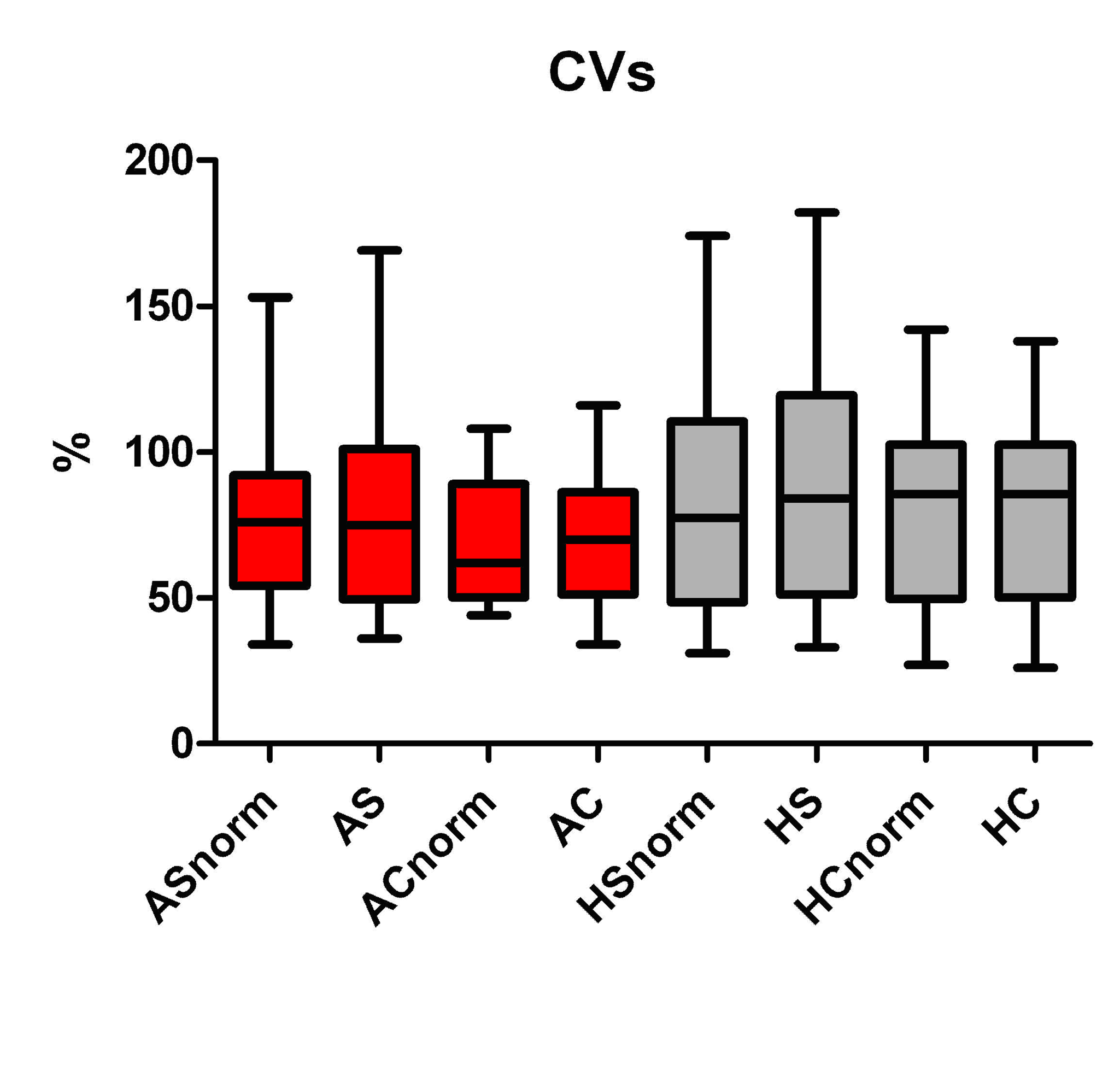

Supplement: Figure S1 — Coefficients of variance (CVs) for all oxylipins before and after BAL-fluid recovery volume normalization (norm). These data support the decision to normalize the oxylipin data with the BAL-fluid recovery volumes due to a decrease in the observed CVs. Abbreviations: AS: Asthmatics following subway air exposure; AC: Asthmatics following control air exposure; HS: Healthy individuals following subway air exposure; HC: Healthy individuals following control air exposure. (TIF) [file pone.0023864.s001.tif]

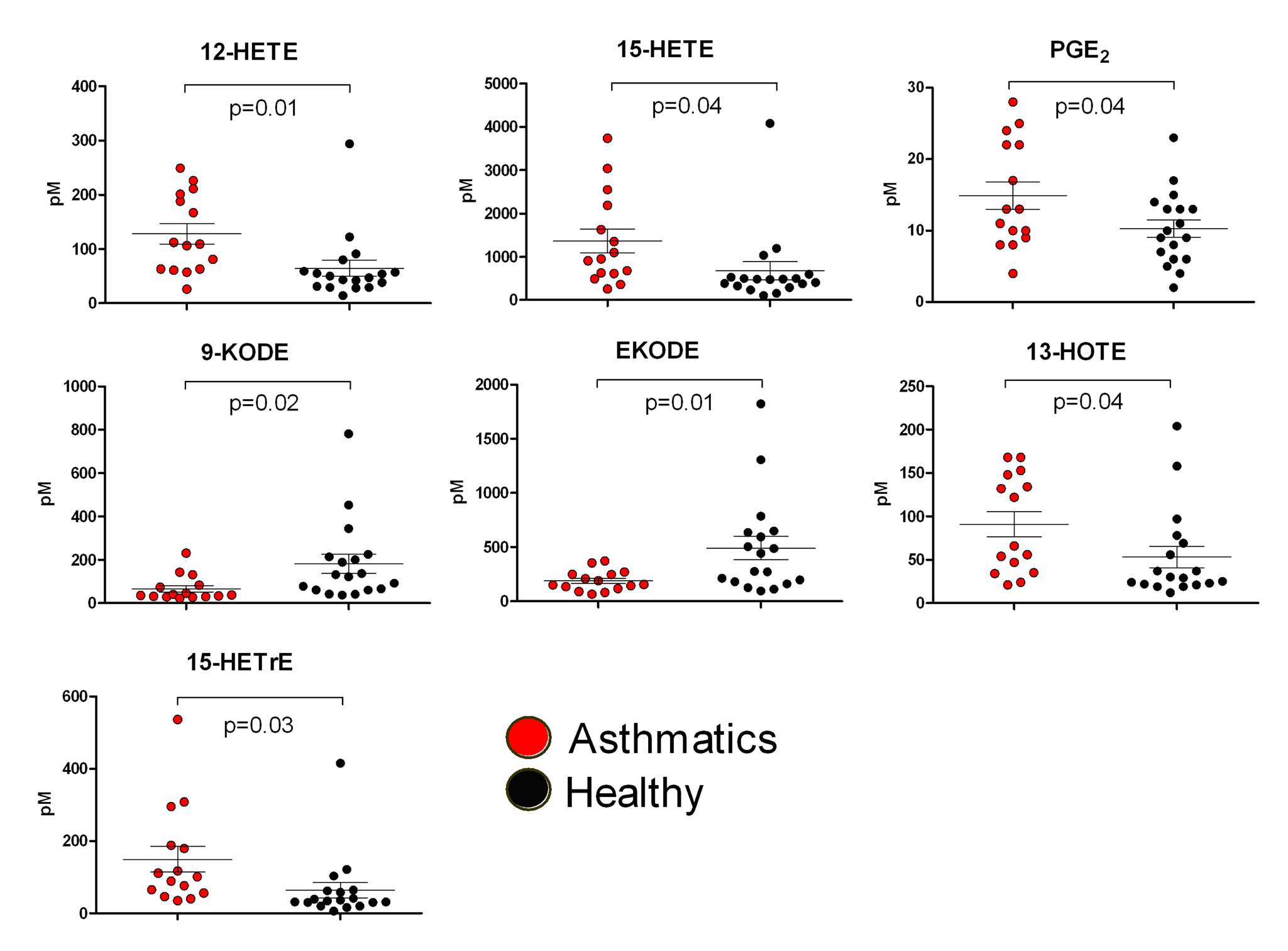

Supplement: Figure S2 — Oxylipin baseline concentrations (pM) of those metabolites indicating significantly different levels between healthy individuals and asthmatics. (TIF) [file pone.0023864.s002.tif]

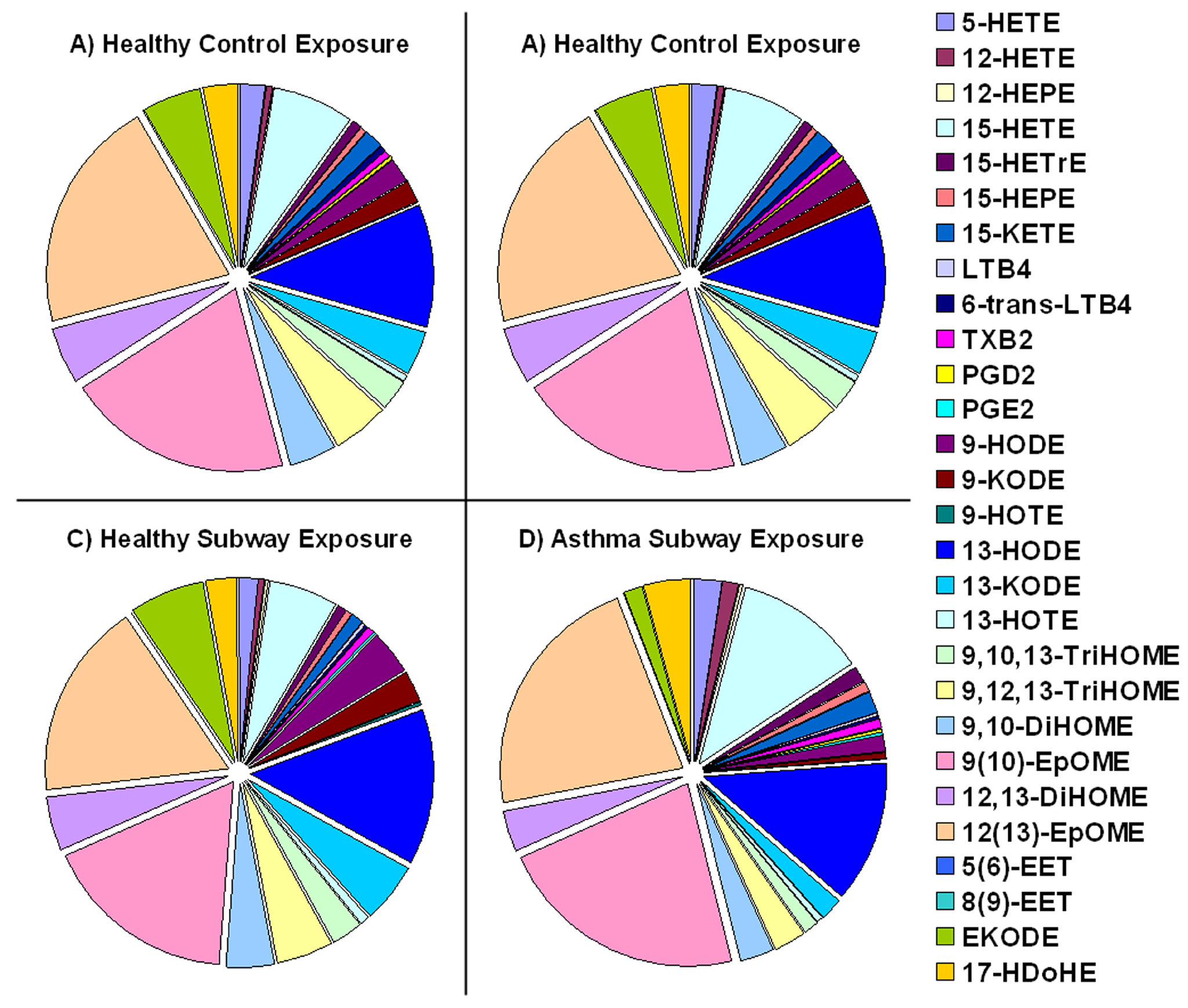

Supplement: Figure S3 — Oxylipin composition in BAL-fluid of the 4 different exposure groups. Oxylipin naming is as provided in Table S1, with data taken from Table 2. (TIF) [file pone.0023864.s003.tif]
